# MicroRNA‐451a acts as tumor suppressor in cutaneous basal cell carcinoma

**DOI:** 10.1002/mgg3.473

**Published:** 2018-09-13

**Authors:** Hui Sun, Pingdong Jiang

**Affiliations:** ^1^ Department of Dermatology Wuxi No.2 People’s Hospital Wuxi, Jiangsu China

**Keywords:** Basal cell carcinoma, K5tTA/TREGLI1, miRNA‐451a, *TBX1*

## Abstract

**Background:**

Basal cell carcinoma (BCC) is the most common type of skin cancer. The underlying mechanism leading to BCC formation is not fully uncovered. The aim of this study was to characterize miRNA‐451a as a novel tumor suppressor in cutaneous BCC.

**Methods:**

We first evaluated miRNA‐451a level in human BCC clinical tissues and inducible BCC mouse model. Then we studied the impact of overexpressing or inhibiting miR‐451a in cell proliferation, colony formation potential, and cell cycle pattern. Next, we employed luciferase reporter assay and western blotting to evaluate *TBX1* as a downstream target of miRNA‐451a. Lastly, we confirmed *TBX1* expressional change in BCC tissues by qPCR.

**Results:**

miRNA‐451a was significantly reduced in human BCC tissues. The downregulation of miRNA‐451a was also confirmed in BCC mouse model. Overexpressing miRNA‐451a in tumor cells markedly suppressed cell growth through G1 cell cycle arrest. However, inhibiting miRNA‐451a in primary cells promoted cell growth and colony formation capacity. *TBX1* (602054) was predicted as a downstream target of miRNA‐451a and this was confirmed by luciferase assay and protein expression. Finally, *TBX1* level was shown upregulated in BCC tissues as inversely to miR451a.

**Conclusion:**

Our studies revealed that miRNA‐451a/*TBX1* axis played a pivotal role in BCC tumorigenesis.

## 1 INTRODUCTION

Basal cell carcinoma (BCC) is the most common type of skin cancer. It makes up about 75%–80% of non‐melanoma skin cancer. In US, more than four million cases of BCC are reported every year. BCC arises from skin basal cells from a lowest layer of epidermis that lines above dermal and epidermal junction. The lesions look like open sores, red patches, or scars. UV exposure is considered the primary cause of BCC formation. Genetic predisposition may also increase the BCC susceptibility in many individuals (Marzuka & Book, 2015; Rubin, Chen, & Ratner, 2005; Wong, Strange, & Lear, 2003). Hedgehog (HH) pathway is the key event in the initiation of the pathogenesis of BCC. Abnormal activation of HH signaling results in constitutive activation of target genes that drive the oncogenesis of BCC, including *SHH* (600725), *SMO* (601500), *GLI1* (165220), and *GLI2* (165230) (Emmert, Schon, & Haenssle, 2014; Otsuka, Levesque, Dummer, & Kabashima, 2015; Wong & Dlugosz, 2014). Transgenic mouse models have been used to confirm the essential role of HH pathway in BCC formation. For instance, grafting transgenic human skin expressing *SHH* on immune‐deficient nude mice also recapitulates the abnormal skin features found in human BCC lesion. Mice overexpressing human *GLI‐1* in the skin epidermis develop tumors resembling human BCC. *GLI1* and *GLI2* form a positive feedback loop in HH‐mediated BCC oncogenesis (Kasper et al., 2011; Nilsson et al., 2000).

MicroRNAs (miRNA) are small single stranded noncoding RNAs of usually 22 nucleotides that play a role in regulating gene expression. They bind to the 3′UTR of their target mRNAs to inhibit the protein expression by destabilizing the mRNA or silencing translational machinery. Usually the 3′UTR of one gene can be targeted by multiple miRNA. Reversely, one miRNA can target many gene targets. miRNA have been found to be involved in a wide array of biological processes including cell proliferation, differentiation, migration, and apoptosis. They have also been reported to play essential roles in many physiological or pathological processes including immune response, neurogenesis, muscle development, and cancers. Many miRNAs have been identified in a broad range of cancers. They can serve as diagnostic markers or potential therapeutic targets (Hammond, 2015; Hayes, Peruzzi, & Lawler, 2014; Li & Rana, 2014).

The regulatory role of miRNAs in BCC pathogenesis is not yet fully understood. One recent study has described the miRNAs expression profile in BCC, which has revealed the potential function of several miRNA candidates in BCC tumorigenesis. For instance, miRNA‐203 has been characterized as a novel tumor suppressor in BCC model. It is downregulated in BCC and its downregulation leads to the activation of HH pathway that eventually contributes to BCC formation (Sonkoly et al., 2012).

miR‐451a was first reported in the regulation of MDR1/P‐glycoprotein in multidrug‐resistant cancer cells. It works together with miR‐27a to activate P‐glycoprotein (Guo et al., 2016). Later studies have revealed miR‐451a carries tumor suppressive role in multiple cancers. For instance, repression of miR‐451a is essential step for T‐cell acute lymphoblastic leukemia. The downregulation of miR‐451a leads to the activation of NOTCH1 signaling pathway that eventually drives oncogenesis (Li, Sanda, Look, Novina, & von Boehmer, 2011). miR‐451a has also been reported as tumor suppressor in human glioma. It impacts glioblastoma cell apoptosis, proliferation, and invasion through targeting PI3K/AKT signaling pathway (Du et al., 2015).

Our study was to characterize the potential role of miR‐451a in BCC pathogenesis. We first analyzed the expressional level of miR‐451a in BCC clinical tissues. And then we monitored its change in mouse model induced with BCC lesion. Subsequently we proceeded to evaluate the functional implication of downregulating miR‐451a in skin cells. Next, we tested the impact of overexpressing miR‐451a in BCC cell line. Lastly, we identified and characterized one potential downstream target of miR‐451a in BCC model and revealed the regulatory mechanism of miR‐451a in BCC pathogenesis.

## 2 MATERIALS AND METHODS

### 2.1 Clinical BCC samples

Tumor tissues were harvested from 22 BCC patients given with informed consents. The tumors were in either Stage I or II as evaluated by dermatologists. The adjacent non‐tumor tissues were also harvested from these patients. The tissues were kept in −80°C until further experiment. The study protocol was approved by the hospital ethics committee.

### 2.2 BCC mouse model

Mice expressing tetracycline‐dependent transactivator regulated by *KRT5* (148040) promoter (*k5tTA*) were crossed with mice expressing human *GLI1* regulated by tetracycline response element (*TREGLI1*) to generate BCC mouse model (*k5tTA/TREGLI1*). Skin BCC lesions driven by *GLI1* expression were induced by withdrawal of doxycycline (1 mg/ml) from the drinking water at week 3 after birth. BCC tissues or non‐tumor tissues from the matched sites of control mice were harvested for miRNA‐451a or *TBX1* gene expression analysis.

### 2.3 Gene expression analysis

Total miRNA or mRNA from sample tissues or cells was harvested with miRNeasy mini kit from Qiagen. miRNA‐451a level was measured with miRCURY™ LNA™ Universal RT microRNA PCR kit from Exiqon. RUN6B RNA was used as normalization control. *TBX1* (NG_009229.1) gene expression was analyzed with TaqMan^®^ Assay kit from Thermo Fisher.

miRNA‐451a‐5p: 5′‐AAAAAAACCGTTACCATTACTGAGTT‐3′
*TBX1* F: 5′‐CTGACCAATAACCTGCTGGATGA‐3′
*TBX1* R: 5′‐GGCTGATATCTGTGCATGGAGTT‐3′

### 2.4 Mammalian cell culture

Human primary adult keratinocytes (PCS‐200‐011) and BCC cells (TE 354.T) were ordered from ATCC. They were cultured according to the manufacturer's instruction.

### 2.5 Antibodies and reagents

Antibodies against human Tbx1 (ab18530) and Gapdh (ab9485) were from Abcam. miRNA‐451a mimic and its scramble control were ordered from Sigma‐Aldrich. miRNA‐451a inhibitor and its negative control were synthesized by Dharmacon.

### 2.6 Cell transfection

Primary or tumor cells were plated at and grown at 5% CO_2_/37°C until about 80% confluence. The miRNA mimic or inhibitor (100 nM) was transfected with Lipofectamine 3,000 according to the manufacturer's instruction.

### 2.7 Cell proliferation assay

After transfected with either miRNA mimic or inhibitor for 48 hr, cells were seeded in 96‐well plates at 5,000 cells/well and cultured at 37 Co/5% CO_2_. Cell proliferation at every 24 hr was measured using Cell Counting Kit‐8 following the manufacturer's instruction. The plates were read at 450 nm. Each group was run in triplicate.

### 2.8 Cell cycle assay

The cells 48 hr after seeding were fixed in 70% ethanol and stained with FxCycle Violet Stain kit from ThermoFisher. The cell cycle analysis was carried out by BD FACSCalibur. Each group was performed in triplicate.

### 2.9 Colony formation assay

Cells were seeded in culture plate at 100 cells/cm^2^. After 2 weeks of incubation, the cell colonies were stained with Giemsa dye. The colonies with a minimum number of 50 cells were considered positive. Each group was run in triplicate.

### 2.10 Luciferase reporter assay

The 3′‐UTR region containing miR‐451a targeting site of *TBX1* or the mutated sequence were cloned downstream of the firefly luciferase gene in pMIR‐REPORT vectors. The constructed reporter genes were then co‐transfected with Renilla Luciferase control (pRL‐SV40, Promega, WI, USA) in cells expressing either miR‐451a mimics or negative control. The luciferase activation was determined with Dual Luciferase Reporter Assay (Promega) according to manufacturer's instruction.

### 2.11 Western blotting analysis

Total protein amount of cell lysate was determined using Bradford assay. About 30 μg protein was resolved in 12% SDS‐PAGE and then transferred onto nitrocellulose membrane. After blocked with 5% milk, the membrane was incubated with primary antibody (1:1,000) at 4°C for overnight. After washing with PBST, the membrane was incubated with a 2nd antibody (1:5,000) at RT for 1 hr. The target protein expression was visualized using SuperSignal West Pico PLUS chemiluminescent kit.

### 2.12 Statistical analysis

Results were presented as mean ± standard deviation (*SD*) from at least three independent experiments. Data analysis was performed with paired or unpaired Student's *t* test. One‐way ANOVA with multiple comparisons was applied on the experiments with more than two groups. Only *p* value less than 0.05 was considered significant.

## 3 RESULTS

### 3.1 miRNA‐451a is downregulated in BCC tumor

**Figure 1:**
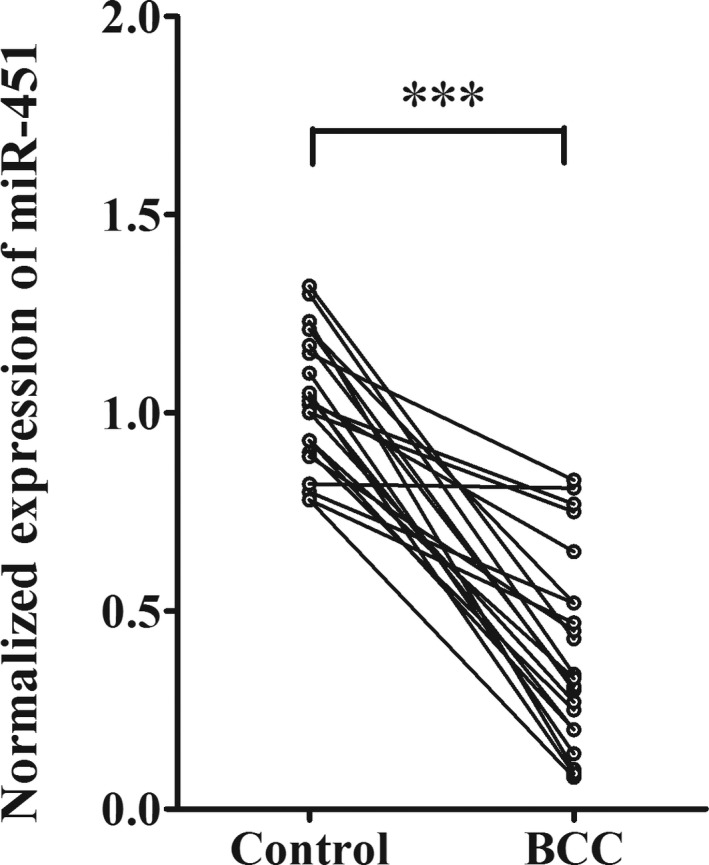
miRNA‐451a is downregulated in BCC tissues. The total miRNAs were extracted from the tissues harvested from BCC sites and the adjacent non‐tumor sites of 22 patients. The expressional level of miRNA‐451a from the samples was determined by real‐time PCR in experimental triplicate. The comparison of miRNA‐451a expression in between tumor and non‐tumor tissues was analyzed by paired Student *t* test (*p < 0.05 or **p < 0.01 or ***p < 0.001, significantly different from control group)

miRNA‐451a has been studied as tumor suppressor in multiple types of cancers (Pan, Wang, & Wang, 2013). To establish its association with skin basal cell carcinoma, we examined miRNA‐451a expressional level in BCC tissues from 22 patients. As comparison, we also measured miRNA‐451a level in the adjacent normal tissue from the same donor. As shown in Figure 1, miRNA‐451a expressed significantly lower in BCC tissues than the matched normal tissues. The fold change varied among patients and ranged from about twofolds to more than 10‐fold decrease. The observation based on the clinical samples strongly suggested a link between downregulation of miRNA‐451a and BCC.

### 3.2 miRNA‐451a is downregulated during BCC tumorigenesis

**Figure 2:**
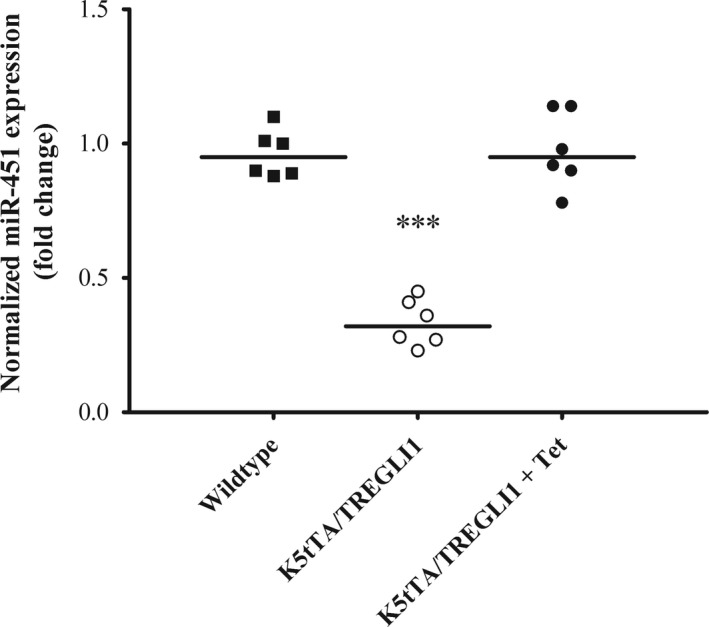
miRNA‐451a is downregulated in BCC mouse model. BCC skin lesions were induced in K5tTA/TREGLI1 mice (*n* = 6 for each group) by removal of doxycycline from drinking water. The BCC tissues or control tissues from either wild‐type mice or K5tTA/TREGLI1 without tumor induction were harvested. miRNA‐451a tissue expression was determined by real‐time PCR (*n* = 6, from each group). The statistical difference was measured by student *t*‐test as compared with wild‐type control (*p < 0.05 or **p < 0.01 or ***p < 0.001, significantly different from control group)

To examine whether the change of miRNA‐451a expression was implicated in BCC tumorigenesis, we employed a relevant BCC mouse model, *K5tTA/TREGLI1*, to monitor miRNA‐451a expressional response in tumor formation. In *K5tTA/TREGLI1* mice, the expression of BCC oncogene *GLI1* was under control of tetracycline response element, while tetracycline transactivator was regulated by cytokeraine 5 promoter and expressed in the skin only. Therefore, *GLI1* expression was suppressed in the presence of doxycycline in the drinking water. To induce BCC lesions, we induced *GLI1* expression in mice of about 3‐week old by removing doxycycline from drinking water. About 6 weeks later, keratinocytes from the tumor lesions were harvested for miR‐451a expression study. As comparison, the keratinocytes from the same sites on both wild‐type or *K5tTA/TREGLI1* mice without *GLI1* induction were also collected. As shown in Figure 2, miR‐451a level was markedly suppressed in the skin lesions overexpressing *GLI1* as compared with wild‐type samples. The expression level was comparable between *K5tTA/TREGLI1* without BCC induction and wild‐type control, which excluded that the differential expression of miR‐451a was due to the genetic background of the mice. This result resembled the pattern we observed in human clinical sample. Taken together, the data revealed a strong link between miR‐451a and BCC tumor formation.

### 3.3 Inhibiting miRNA‐451a in primary keratinocytes induces cell growth

**Figure 3:**
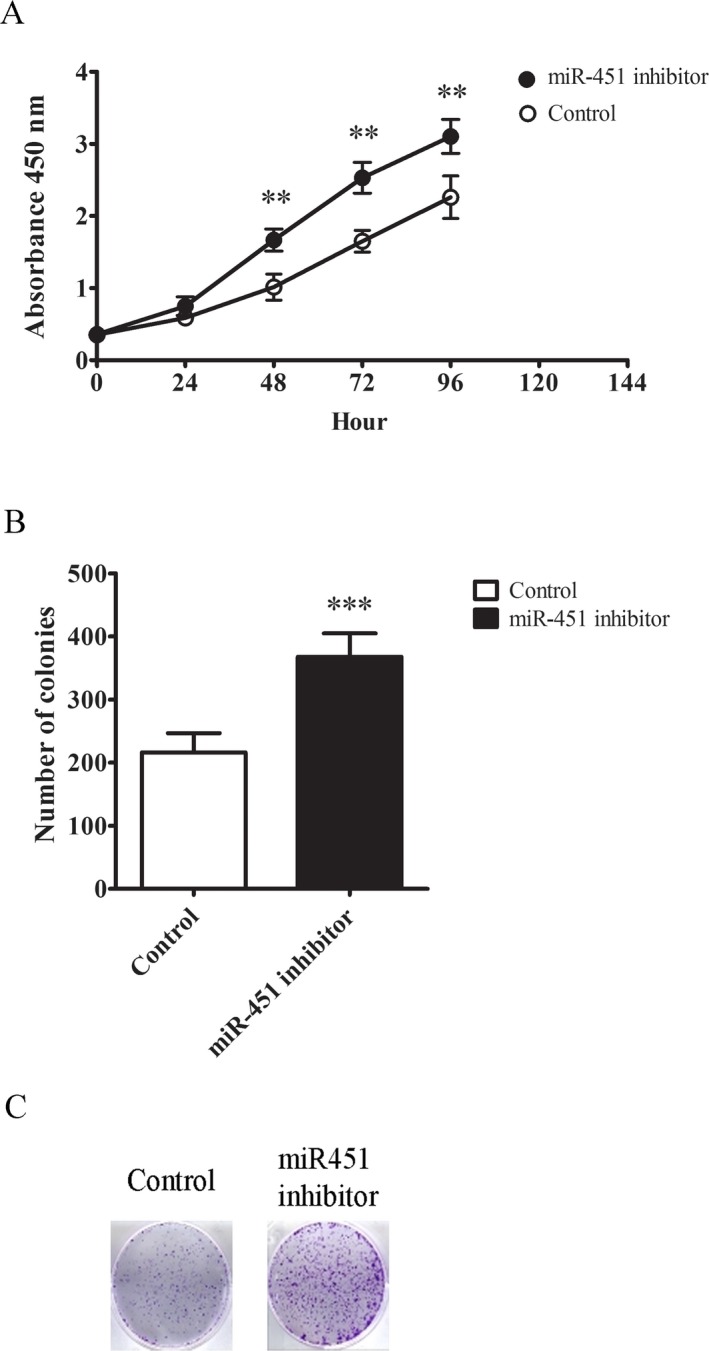
Inhibiting miRNA‐451a in primary keratinocytes promotes cell growth. (a) Cell proliferation curves of primary keratinocytes treated with either miRNA‐451a inhibitor or negative control. Forty‐eight hours after treatment, the cells were plated and their growth rates were monitored every 24 hr for 4 days with Cell Counting Kit‐8. (b) Colony formation assay result of cells treated with either miRNA‐451a inhibitor or negative control. The cells were plated in the well and the number of colonies (with at least 50 cells) were counted 14 days later. (c) Representative graph showing the colony formation pattern of each cell line group. The data were presented as means ± *SD* (*p < 0.05, **p < 0.01, ***p < 0.001, significantly different from control group)

Next, we proceeded to test whether miR‐451a played a role in BCC tumorigenesis. Primary keratinocytes were transfected with miR‐451a inhibitor or negative control. Forty‐eight hours after transfection, the cells were replated and their proliferation rate was monitored under OD 450 nm for 4 days. As shown (Figure 3a), keratinocytes treated with miR‐451a inhibitor showed significantly higher growth rate than the control cells. The difference was first observed 48 hr after seeding. We also measured the colony formation potential of the cells. This assay served as an indicative of tumor formation capacity of cell subjects. As expected, cells transfected with miR‐451a inhibitor showed more colonies formation than the control cells, with about twofold increase (Figure 3b). The representative staining data were shown to confirm the counting (Figure 3c). Therefore, our results here suggested miR‐451a may carry tumor suppressive function in skin.

### 3.4 Overexpressing miRNA‐451a reduces BCC cell proliferation

**Figure 4:**
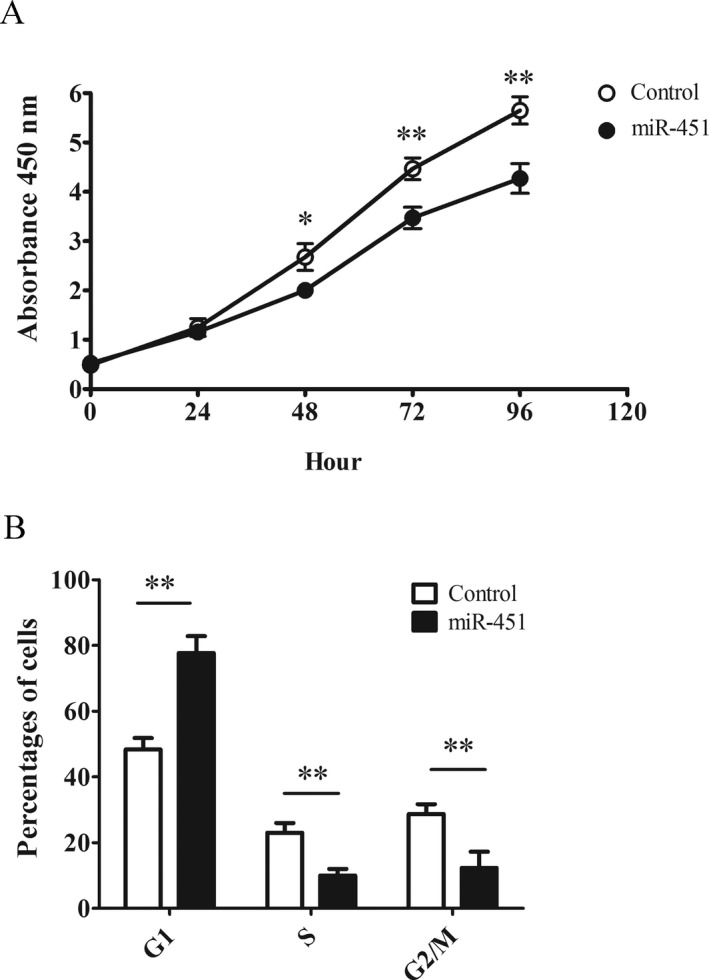
Overexpression of miRNA‐451a reduces TE 354.T cells proliferation through cell cycle arrest. (a) Cell proliferation curves of BCC cells transfected with either miRNA‐451a or negative control. Forty‐eight hours after treatment, the cells were plated and their growth rates were monitored every 24 hr for 4 days with Cell Counting Kit‐8. (b) The cell cycle analysis of the cells treated with miRNA‐451a mimic or negative control. The cells were harvested and stained with PI. The cell cycle distribution of the cell population was quantified by DNA content analysis. The data were presented as means ± *SD* (*p < 0.05, **p < 0.01, ***p < 0.001, significantly different from control group)

To further confirm miR‐451a as a tumor suppressor in BCC, we studied the impact of overexpressing miR‐451a in BCC tumor cell line, TE 354.T. We transfected the tumor cells with miR‐451a and measured cell growth 48 hr later. Interestingly, miR‐451a expression markedly slowed down the cancer cell proliferation as observed from 48 to 96 hr post seeding (Figure 4a). The anti‐proliferating effect from miR‐451a in TE354.T cells suggested it as tumor suppressor in BCC. Next, we profiled cell cycle pattern in the cells overexpressing miR‐451a with cells expressing scramble miRNA as control. As shown (Figure 4b), 50% of the control cells population were in G1 phase, along with about 20% or 30% in S or G2/M phase, respectively. In comparison, nearly 80% of the cells expressing miR‐451a were arrested in G1, with only about 10% entering S or G2/M phase, respectively. The impact in cell cycle pattern indicated miR‐451a played a regulatory role in cell cycle control that subsequently suppressed tumor cells proliferation.

### 3.5 TBX1 is downstream target of miRNA‐451a

**Figure 5:**
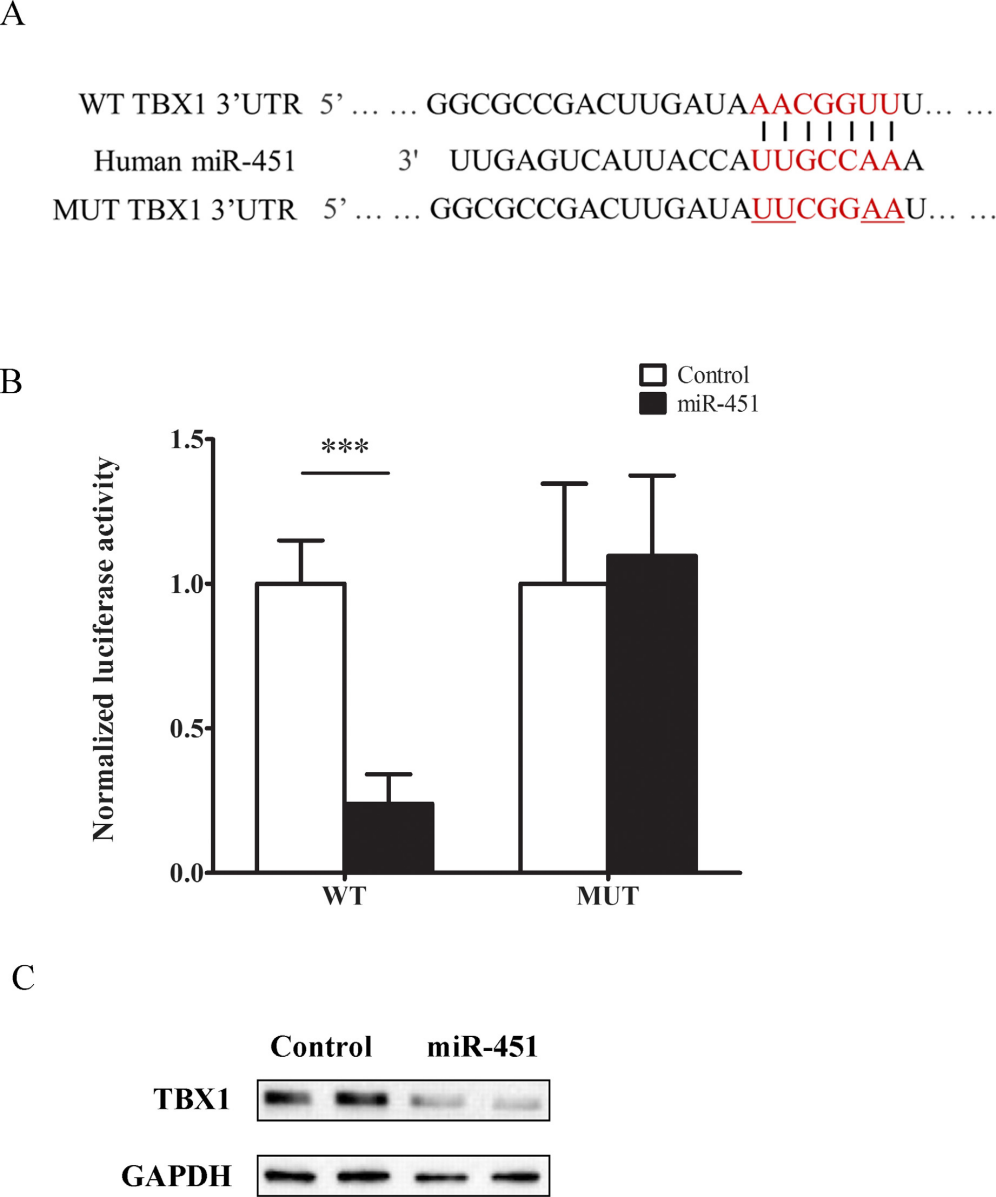
*TBX1* is downstream target of miRNA‐451a. (a) The conserved miR‐451a targeting site was shown in the 3′UTR of *TBX1 *(NG_009229.1). The lower sequence showed the site mutations introduced to the binding site of 3′UTR. (b) Cells expressing luciferase reporter genes (WT: 3′UTR of *TBX1 *vs MUT: mutated 3′UTR of *TBX1*) were transfected with either miR‐451a mimic or negative control. After 48 hr, the luciferase activities were quantified with dual luciferase kit (Promega). The data were presented as means ± *SD* with triplicate in each group (*p < 0.05, **p < 0.01, ***p < 0.001, significantly different from control group). (c) Western blot analysis of *TBX1* expression in cells transfected with either miR‐451a mimic or control miRNA. The result was representative of three independent experiments

**Figure 6:**
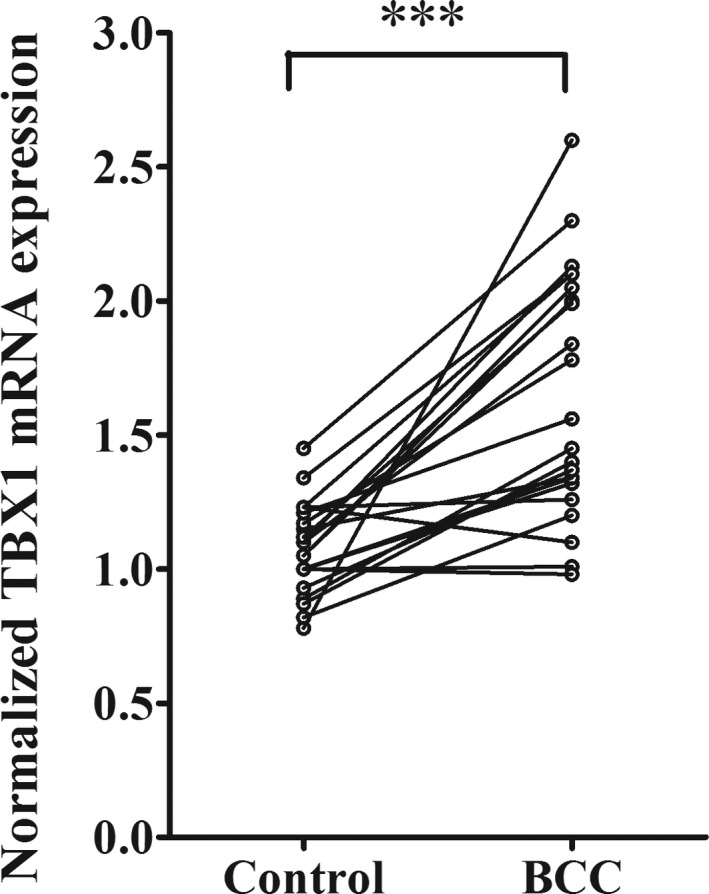
*TBX1* is downregulated in BCC tissues. The total mRNAs were extracted from the tissues harvested from BCC sites and the adjacent non‐tumor sites of 22 patients. The expressional level of *TBX1* in the samples was determined by real‐time PCR in experimental triplicate. The differential expression of *TBX* in between tumor and non‐tumor tissues was compared by paired Student *t*‐test (*p < 0.05 or **p < 0.01 or ***p < 0.001, significantly different from control group)

To provide more mechanistic understanding on the tumor suppressor role of miR‐451a in BCC, we proceeded to identify its downstream target involved in BCC parthenogenesis. We firstly predicted miR‐451a downstream targets in TargetScanHuman 7.2 database (Agarwal, Bell, Nam, & Bartel, 2015). Among the top list, one of the potential targets was *TBX1*, which belongs to a family of transcriptional factors involved in cell proliferation and organ development. Many studies have reported *TBX* genes carry essential roles in tumorigenesis and progression in multiple cancer types. miR‐451a targeting site was found conservatively in *TBX1* 3′UTR (Figure 5a). To test this target candidate, we employed luciferase gene report system. In WT system, 3′UTR of *TBX1* containing miR‐451a binding site was cloned into downstream of luciferase reporter gene. In MUT system, similar 3′UTR sequence containing mutated miR‐451a targeting site was conjugated to the downstream of reporter gene as shown in Figure 5a. First, we co‐transfected WT reporter genes into primary keratinocytes with either miR‐451a or control miRNA. As expected, miR‐451a expression markedly suppressed the expression and activity of WT luciferase gene as compared with control miRNA (Figure 5b). Next, we co‐expressed MUT reporter genes into the same cells with either miR‐451a or negative control. Interestingly, the mutation on miR‐451a binding site of *TBX1* 3′UTR completely abolished miR‐451a suppressing effect on luciferase gene (Figure 5b), indicating the interaction between miR‐451a and 3′UTR of *TBX1* was interrupted. Furthermore, we moved on to study the regulatory effect of miR‐451a on *TBX1* protein expression. Primary keratinocytes were transfected with either miR‐451a or control miRNA and harvested for protein analysis 48 hr later. As shown (Figure 5c), the expression of miR‐451a markedly reduced* TBX1* expressional level in keratinocytes, which confirmed that *TBX1* was downstream target of miR‐451a in cells. Lastly, we would like to validate clinical relevance of *TBX1*, as a downstream target of miR‐451a, in BCC. We measured *TBX1* gene expression in BCC tissues from 22 patients that showed overall downregulation of miR‐451a in the previous experiment. Interestingly, in comparison with normal control tissues, *TBX1* mRNA level was upregulated significantly in these BCC samples as inversely correlated with miR‐451a expression (Figure 6). Taken together, these results strongly suggested *TBX1* was a direct target of miR‐451a and implicated in BCC pathogenesis.

## 4 DISCUSSION

This is first study in the field to discover miRNA‐451a as a novel tumor suppressor for BCC. We have showed miRNA‐451a level decreases significantly in 22 BCC tissues. This is also evidenced by the reduction of miRNA‐451a in BCC mouse model. Knocking down miRNA‐451a in primary keratinocytes, which resembles the expression change in BCC condition, promotes tumor cell‐like phenotypes including increased cell growth and colony formation capacity. In contrast, restoring miRNA‐451a expression in BCC cell line slows down cell proliferation through inducing cell cycle arrest. This strongly supports a therapeutic potential of targeting miRNA‐451a in BCC. In the study, we also investigate further on the mechanistic regulation of miRNA‐451a in BCC tumorigenesis. We confirm *TBX1* is direct target of miR451a in cells and 451aits level is upregulated in BCC tissues451a. This strongly implies that miRNA‐451a/*TBX1* axis may play a critical part in BCC formation.

miRNA‐451a as tumor suppressor has been reported in many forms of cancers in previous studies. But the regulatory mechanism or targets are tissue specific. Wang et al. described that miRNA‐451a plays a key function in head and neck squamous cell carcinoma (HNSCC) (Wang et al., 2015). miRNA‐451a expression is repressed in both HNSCC tissue and cell line. The forced expression of miRNA‐451a leads to the downregulation of c‐Myc oncogene that is suggested to contribute its tumor suppressive role in HNSCC. In glioma cells, miRNA‐451a can regulate glucose transporters such as *GLUT1* on the cell membrane to downregulate glucose metabolism and prevent energy supply to cancer cells (Guo et al., 2016). In the same study, *CAB39* (612174) has been identified as a target of miRNA‐451a. The downregulation *CAB39* by miRNA‐451a inhibits PI3K/Akt pathway in glioma cells. Furthermore, Huang et al. have provided a comprehensive study to elucidate the mechanistic role of miRNA‐451a in the progression and metastasis of HCC (Huang et al., 2015). In this study, loss‐ and gain‐of‐function experiments clearly demonstrated that miR‐451a can induce G0/G1 arrest and inhibited cell growth in HCC cells. Importantly, miR‐451a can inhibit epithelial–mesenchymal transition process, which suggests it as potential metastasis inhibitor of HCC. The tumor suppressive role of miR‐451a in HCC is suggested at least partially through targeting *MYC* (190080), the same regulatory target proposed in HNSCC. Similarly, our characterization study in BCC model also demonstrates miR‐451a overexpression can induce cell cycle arrest in skin tumor cells. Interestingly, we identified a different downstream target *TBX1* that may be accountable for cell cycle regulation in BCC model.

*TBX1* is an important transcriptional factor in T‐box family that plays a regulatory role in tissue development. It is highly expressed in hair follicle stem cells (Trempus et al., 2011). The downregulation of *TBX1* in mouse skin may trigger skin tumor development. In the same study, it has been shown that *TBX1* expression in tumor cells significantly decreases cell growth and colony formation. But another study reveals the expression level of *TBX1* affects heart development. It is detected in cardiac progenitor cells and binds to serum response factor to support cardiac progenitor cells proliferation and suppress their differentiation (Chen, Fulcoli, Tang, & Baldini, 2009). Similarly,* TBX1 *has been shown to be required for lymphatic vessel development (Chen et al., 2010). Conditional knockout of *TBX1* in endothelial cells results in lymphangiogenesis defects. It promotes the lymphatic vessels growth by regulating Vegfr3 expression. Therefore, *TBX1* can function as either positive or negative factor in regulating cell growth or tissue development, depending on the regulatory network or tissue specific pathway it is involved. Our preliminary study suggests *TBX1* may carry oncogenic function in BCC model. We have validated it as a direct target of miRNA‐451a and its level is significantly upregulated in BCC tumor tissues. More characterization studies are needed in future to elucidate the role of *TBX1* in BCC. It will be interesting to validate whether *TBX1* is a new biomarker associated with the occurrence or progression of BCC. Or *TBX1* plays an essential part to promote BCC tumorigenesis and can be used as therapeutic target to treat BCC. For instance, miRNA‐451a expression can lead to cell cycle arrest, which may be mediated through *TBX1 *downregulation. It will be intriguing to check whether knocking down *TBX1* in BCC cells will have an impact on cell cycle pattern. In a recent study, dysregulated* TBX1* level has been associated with parathyroid tumor model (Verdelli et al., 2017). Silencing* TBX1* in cells induces cell cycle arrest in G0/G1 phase, which supports our hypothesis that* TBX1 *may regulate cell growth through cell cycle control.

Although the mortality is low in BCC as it rarely metastasizes. But the development of malignance if unmanaged may increase the risk of developing other lethal forms of skin cancers such as melanoma. Treatments for BCC can be nonsurgical or surgical. The common topical treatments for BCC include 5% fluorouracil and 5% imiquimod. They are effective for superficial BCC but are not specific and associated with increased incidence of side effects if being applied frequently (Alter, Hillen, Leiter, Sachse, & Gutzmer, 2015; Clark, Furniss, & Mackay‐Wiggan, 2014). Therefore, a new target‐based therapy for BCC could lead to a more effective and safer solution. Our study has discovered miRNA‐451a plays a tumor suppressive role in BCC. miRNA‐451a and its downstream target such as *TBX1* may serve as intervention point to manage BCC malignance. The immediate next step to test this hypothesis will be to study the therapeutic effect of topically application of miRNA‐451a in mouse BCC model, which will provide more insightful view to predict the clinical effect of targeting miRNA‐451a or its regulatory circuit in BCC treatment.

## CONFLICT OF INTEREST

The authors declare that they have no conflict of interest.
